# Effect of S-Allyl-L-Cysteine on Nitric Oxide and Cadmium Processes in Rice (*Oryza sativa* L. sp. Zhongzao35) Seedlings

**DOI:** 10.3390/toxics12110805

**Published:** 2024-11-07

**Authors:** Xingyu Huo, Changrong Wang, Yongchun Huang, Weiyong Kong, Xiaoli Wang

**Affiliations:** Agro-Environmental Protection Institute, Ministry of Agriculture and Rural Affairs, Tianjin 300191, China; 82101225357@caas.cn (X.H.); wangchangrong@caas.cn (C.W.); 82101215310@caas.cn (W.K.); 82101205249@caas.cn (X.W.)

**Keywords:** S-allyl-L-cysteine, rice, cadmium, nitric oxide, transport

## Abstract

Nitric oxide (NO) is an important signaling molecule involved in regulating plant processes to cope with abiotic stress. S-allyl-L-cysteine (SAC) is known to induce NO synthesis in animals. However, it is unknown whether SAC can trigger NO biosynthesis, regulate Cd transport, or alleviate Cd stress in plants. After being sprayed with 0.2 mM SAC, rice seedlings had a NO content that was 1.8 times higher than that of the control (ctrl) group at the ninth hour, which then gradually decreased. The expressions of Cd uptake and transport genes in the roots (including OsNRAMP5, OsNRAMP1, and OsHMA2) were markedly downregulated by 27.2%, 24.8%, and 49.1%, respectively, 72 h after SAC spraying treatment. The Cd content in seedling roots’ cell wall (CW) components significantly increased by 43.5% compared to that of the ctrl group. The Cd content in the shoots and roots decreased by 49.0% and 29.8%, respectively. Cd stress in the seedlings was also substantially alleviated. In conclusion, spraying rice seedlings with SAC triggered an increase in NO synthesis, regulated the expression of genes related to Cd transport, increased Cd fixation in the root CW components, and reduced Cd accumulation in the roots and shoots.

## 1. Introduction

Industrial and agricultural activities are the primary contributors to the constant release of toxic cadmium (Cd) into the natural environment [1]. Compared with other heavy metal elements, Cd exhibits higher mobility in the soil and readily accumulates in the edible parts of crops [2]. Upon entry into the human body through the food chain, Cd continuously accumulates in the liver, kidneys, and other organs, posing potential risks to human health [3]. In 1993, the International Agency for Research on Cancer (IRAC) defined Cd as a primary carcinogen [4]. In China, rice has become the main source of Cd intake in people, accounting for 56% of the total Cd intake [5]. Due to the toxic effects of Cd on the human body, the Cd concentration in rice grains should be below 0.20 mg·kg^−1^ according to the standard [6] describing China’s maximum allowable levels of contaminants in food to ensure the safety of rice consumption. Therefore, developing agronomic measures that regulate Cd transport in rice and reduce accumulation in grains is important for protecting human health.

Rice lacks specific Cd transporters as Cd is a nonessential metal mineral element for plants. Instead, the uptake and transmembrane transport of Cd mainly depend on Mn, Fe, Zn, and other metal cation transporters [7]. Ishimaru et al. [8] reported that Cd mainly enters rice roots through the Mn transporter OsNRAMP5. In rice with OsNRAMP5 knockout, the Mn and Cd contents in rice grains were significantly reduced [9]. In addition, Chang et al. [10] reported that the OsNRAMP1 transporter is associated with Cd absorption. Upon entering the roots, a portion of Cd is transported into vacuoles by the OsHMA3 transporter [11]. This process is an important pathway as it reduces the effect of Cd stress on rice. Takahashi et al. [12] demonstrated that Cd is mainly transported to rice shoots through the Zn transporter OsHMA2. Inactivation of this transporter significantly decreased the Cd content in the shoots of rice seedlings [13]. The Cd in rice grains is mainly present in the vegetative organs, especially in the flag leaves and nodes [14]. Therefore, regulating the expression of Cd absorption and transport genes reduces the Cd accumulation in vegetative organs, thereby helping decrease the Cd content of rice grains.

Plant signaling regulates the expression of functional genes and various physiological processes related to plant development. Nitric oxide (NO) is an important plant signaling molecule that regulates many important physiological processes, such as seed germination, stomatal closure, and coping with abiotic stress [15]. In recent years, many reports have shown that both endogenous and exogenous NO molecules can participate in regulating heavy metal transport in rice. Huang et al. [16] showed that adding melatonin to the culture medium induces an increase in the NO content in the roots of rice seedlings, downregulates the expression levels of Cd-transport-related genes, and reduces the Cd content in the roots and shoots. Pan et al. [17] reported that NO signaling molecules are involved in the regulation of cell wall (CW) biosynthesis in rice seedling roots, increasing the lignin content of the root CW to prevent Cd from entering the roots and reducing the Cd content in rice seedlings. Xiong et al. [18] revealed that the addition of exogenous NO donor sodium nitroprusside to a hydroponic solution significantly enhances the tolerance of rice seedlings to Cd, increases the contents of hemicellulose and pectin in the root CW, and decreases the content of Cd in seedling shoots. Singh et al. [19] reported that adding sodium nitroprusside to a hydroponic solution reduces arsenite stress on rice seedlings and the total arsenic content in the shoots. Previous studies have reported that NO can regulate the transport of heavy metals by modulating the apoplastic and symplastic pathways in rice. The use of foliar spraying technology to trigger NO biosynthesis in rice is expected to achieve Cd transport regulation in rice.

The foliar application of certain nutrients, such as mineral elements and small-molecule acids including Si, Mn, Se [20], malic acid, and citric acid [21], has been found to affect Cd accumulation and alleviate Cd toxicity in rice and other plants. The Cd-decreasing mechanism of these measures is mainly related to ion antagonism and chemical chelation. Triggering rice NO synthesis through foliar spraying technology, regulating rice Cd transport, and reducing Cd content is a new approach to controlling Cd pollution in rice. Studies found that the natural organic sulfur compound, S-allyl-L-cysteine (SAC), derived from garlic, can induce NO synthesis in animals [22]. Moreover, reports indicate that SAC is one of the most abundant water-soluble organic sulfur compounds with antioxidant and anticancer activities in aged garlic [23]. SAC can effectively remove various harmful reactive oxygen species (ROS), such as hydroxy radicals, peroxide radicals, and singlet oxygen [24]. As an important heavy metal antidote, SAC can pass through the cell membrane to reduce the intracellular toxicity of heavy metal ions [25]. A mature and stable process has been established for extracting SAC from garlic, as it is a highly promising natural product [26]. In addition, chemists have developed a chemical synthesis process for SAC, which utilizes a one-step reaction of cysteine and allyl bromide to obtain high-purity SAC, significantly reducing production costs [27]. However, there is still no report on whether SAC can trigger NO biosynthesis, regulate Cd transport, and reduce plant oxidative damage.

In the present study, rice (*Oryza sativa* L. sp. Zhongzao35) plants were sprayed three times with SAC at the seedling stage, with the aim of investigating the effect of foliar spraying with SAC on Cd accumulation in the shoots and roots of rice seedlings and on Cd stress alleviation in rice seedlings, as well as the potential mechanism underlying the reductions in Cd uptake and transport to the shoots of rice seedlings induced by spraying SAC.

## 2. Materials and Methods

### 2.1. Plant Materials and Growth Conditions

Rice (*Oryza sativa* L. sp. Zhongzao35) seeds were treated with a 5% (*w*/*v*) aqueous H_2_O_2_ solution for 20 min, thoroughly washed five times with deionized water, and then immersed in deionized water for 24 h at 28 °C. The seeds were germinated in deionized water at 28 °C for 4 d. Germinating seeds were transferred to a growth chamber for further cultivation in deionized water. When the seedlings reached the 2-leaf stage, the medium was changed to Hoagland nutrient solution (1/10 of the standard concentration) [28]. The culture medium was changed every 3 d until the seedlings reached the 3-leaf stage.

The rice seedlings were then transferred to a hydroponic tank with five holes, in which 15 rice seedlings were planted. The rice seedlings were cultured in a Hoagland nutrient solution at 1/4 of the standard concentration containing 2.7 μmol·L^−1^ CdCl_2_ (Macklin, Beijing, China). The test group seedlings were cultured for 5 d under Cd^2+^ stress and then sprayed with 0.05, 0.1, 0.2, or 0.4 mmol·L^−1^ of SAC (Macklin, Beijing, China) solution (5.0 mL/hole) a total of three times, with an interval of 3 d between treatments. The blank control (BK) group was sprayed with deionized water without Cd^2+^ or SAC, whereas the control (ctrl) group was sprayed with deionized water by Cd stress. The BK + SAC group was sprayed with 0.2 mmol·L^−1^ of SAC without Cd^2+^. Each spraying treatment was performed in eight hydroponic tanks and was repeated three times. Rice seedlings were harvested 3 d after the last SAC treatment to determine the expression levels of Cd transporter genes and the contents of Cd and other metal nutrient elements.

To investigate the changes in NO content in the roots of the rice seedlings after spraying SAC, four different experimental treatment groups were set up: (1) BK, with neither Cd^2+^ stress nor spraying SAC; (2) BK + 0.2 mmol·L^−1^ of SAC, without Cd^2+^ stress but sprayed with 0.2 mmol·L^−1^ of SAC; (3) ctrl, treated with only 2.7 µmol·L^−1^ of Cd^2+^ and sprayed with deionized water; (4) ctrl + SAC group, sprayed with 0.2 mmol·L^−1^ of SAC and treated with 2.7 µmol·L^−1^ of Cd^2+^. The NO content in the seedling was measured at 0–48 h after spraying SAC.

### 2.2. Measurement of Cd and Mineral (Ca, Mg, Fe, Mn, and Zn) Contents

To determine the concentration of Cd and mineral elements (Ca, Mg, Fe, Mn, and Zn) in the rice seedlings, about 0.25 g of dried and ground plant sample was placed into a digestion tube and digested by soaking overnight in 7 mL of concentrated nitric acid in an electro-thermal digester. Next, the seedlings were heated at 110 °C for 2.5 h and then cooled to 20–25 °C. Subsequently, 1 mL of hydrogen peroxide was added, and the sample was shaken thoroughly while heated at 110 °C for 1.5 h. Finally, the clear and transparent digested sample solution was concentrated at 170 °C to less than 0.5 mL, transferred to a volumetric flask, and diluted to 25.0 mL with deionized water. The concentrations of Cd and mineral elements were determined using inductively coupled plasma mass spectrometry (ICP-MS, Agilent, Santa Clara, CA, USA) [29].

### 2.3. Measurement of MDA Contents

Malondialdehyde (MDA) content was determined as described by Ding et al. [30]. Briefly, rice seedling samples were weighed, placed into a mortar with an appropriate amount of trichloroacetic acid, ground thoroughly, and centrifuged. A small amount of supernatant was mixed with an equal volume of thiobarbituric acid, heated at 95 °C for about 30 min, and then cooled rapidly on ice. Following centrifugation at 10,000× *g* for 20 min, the absorbance at 450, 532, and 600 nm was measured.

### 2.4. Measurement of GSH Contents

Glutathione (GSH) was assayed using an enzymic recycling procedure. Briefly, an appropriate amount of fresh rice seedlings was weighed and ground in an ice bath, to which 5 mL of 5% sulfosalicyclic acid was added, mixed thoroughly, and then centrifuged. Next, NADPH and DTNB were added to the supernatant. After incubation for 3 min at room temperature, the absorbance at 412 nm was determined using a UV–Vis spectrophotometer (Agilent, Santa Clara, CA, USA).

### 2.5. Measurement of Antioxidant Enzyme Activity

The activity of antioxidant enzymes, including superoxide dismutase (SOD) and catalase (CAT), was measured following the protocols of the respective assay kits (Solarbio, Beijing, China) as described by Ding et al. [30]. The antioxidant enzyme activities were defined as described by Ding et al. [30].

### 2.6. Analysis of Cd-Transport-Related Genes

The roots of seedlings grown hydroponically were separately sampled to investigate the relative gene expression levels. The total RNA of fresh tissues was extracted using a Rneasy Plant RNA Mini Kit (Solarbio, Beijing, China) and used to prepare cDNA by reverse transcription using a SuperScript II kit (Invitrogen, Waltham, MA, USA) (Solarbio, Beijing, China). Quantitative real-time PCR (qRT-PCR) was performed using an SYBR Green GoTaq^®®^ qPCRMaster Mix (Solarbio, Beijing, China). The primer sequences for qRT-PCR were as follows: 5′-CATAGTGAAGCTGCCTGAGATC-3′ (forward) and 5′-GATCAAACGCATAGCAGC ATCG-3′ (reverse) for OsHMA2 [30]. 5′-TCCATCCAACCAAACCCGGAAA-3′ (forward) and 5′-TGCCAATGTCCTTCTGTTCCCA-3′ (reverse) for OsHMA3 [31]; 5′-CAGCAGCAGTAAGAGCAAGATG-3′ (forward) and 5′-GTGCTCAGGAAGTACATG TTGAT-3′ (reverse) for OsNRAMP5 [31]; 5′-CGACTAAGCTTAAGAAG CCGCACTAGTATG-3′ (forward) and 5′-CCGGTCTAGAAGGGTACTACACGGGTGGCT-3′ (reverse) for OsNRAMP1 [10]; 5′-GACTCTGGTGATGGTGTCAGC-3′ (forward) and 5′-GGCTGGAAGAGGACCTCAGG-3′ (reverse) for actin, which was used as an internal standard [31]. The relative expression was normalized using the ^ΔΔ^Ct method.

### 2.7. Determination of NO Concentration in Rice Seedling Roots

The NO concentration in the roots of rice seedlings was measured using the method provided with the reagent kit purchased from Solarbio (Beijing, China). NO is easily oxidized to NO^2−^ in the extraction solution, and the generated NO^2−^ reacts with Greiss reagent to produce a purple azo dye. The absorbance of the dye can be quantitatively measured at 540–560 nm [32].

### 2.8. Subcellular Distribution of Cd

Root samples were treated using the method reported by Zhang et al. [33] to determine subcellular distribution. Briefly, frozen rice seedling root samples were homogenized in 5 mM (pH 7.5) Tris-HCl containing 250 mM sucrose and 1.0 mM C_4_H_10_O_2_S_2_. The homogenate was passed through an 80 µm nylon mesh, and the filtrate was collected. The residue retained on the nylon mesh was assigned as the CW fraction. The filtrate was collected and centrifuged at 20,000× *g* for 45 min at 4 °C. The supernatant was assigned as the soluble (S) component and the precipitate as the organelle (O) component. The collected fractions were digested using the procedure described in Section 2.2, and the Cd content of each fraction was determined using ICP-MS (Agilent, Santa Clara, CA, USA).

### 2.9. Determination of Cd Content in Root CW Components of Rice Seedlings

The method described by Wang et al. [34] was used to determine the Cd content in CW components. (1) Pectin extraction: The crude CW fraction was extracted in 5 mL of 0.5% ammonium oxalate buffer (containing 0.1% NaBH_4_), boiled for 1 h, and centrifuged to collect the supernatant liquid. (2) Hemicellulose-1 (HC-1) extraction: The remaining precipitate was extracted at room temperature for 24 h with 5 mL of 4% KOH aqueous solution containing 0.1% NaBH_4_. The resulting solution was centrifuged, and the supernatant was collected. The precipitate was retained for further extraction. (3) Hemicellulose-2 (HC-2) extraction: The precipitate from the previous step was extracted for 24 h with 5 mL of 24% KOH solution containing 0.1% NaBH_4_ at 25 °C. The solution was centrifuged, and the supernatant was collected. (4) Cellulose extraction: After extraction with 24% KOH, the remaining precipitate was freeze-dried.

### 2.10. Determination of Methyl Esterification Degree in Pectin Component of Root CW

The pectin component of the CW fraction was extracted according to the method described in Section 2.9. The content of galacturonic acid (GalA) was determined according to the method described by Liang et al. [35], and the methanol content was determined according to the method described by Zhang et al. [36]. Degree of methyl esterification of pectin (%) = methanol production ÷ GalA content × 100. A one-way analysis of variance was used to assess statistical significance between the treatments. The standard errors were determined from the 8 replicates within each treatment.

### 2.11. Data Analysis

Origin2022 software (Origin Lab, Northampton, MA, USA) was used for data calculation and chart drawing. SPSS22.0 statistical software (IBM, Armonk, NY, USA) was used for one-way analysis of variance (ANOVA), Duncan’s multiple comparison, and significant difference test.

## 3. Results

### 3.1. Effect of SAC on Cd and Mineral Concentrations in Rice Seedlings

**Figure 1:**
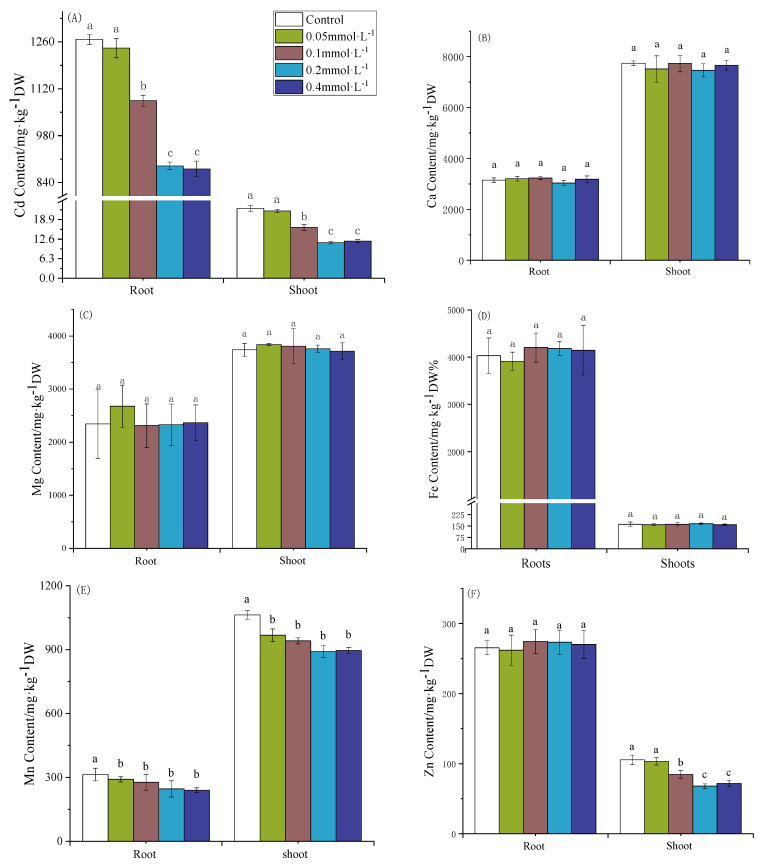
Effect of spraying SAC on Cd and other mineral element concentrations in rice seedlings. (**A**) Cd, (**B**) Ca, (**C**) Mg, (**D**) Fe, (**E**) Mn, and (**F**) Zn contents in rice seedlings (roots and shoots). a, b and c represent the significance of the differences in each treatment.

To investigate the effects of spraying SAC on the transport of Cd and other minerals, ICP-MS was used to determine the concentrations of Cd and other minerals in the shoots and roots of the rice seedlings. As shown in Figure 1A, higher concentrations of Cd accumulated in both the shoots and roots of the rice seedlings upon treatment with 2.7 µmol·L^−1^ of CdCl_2_ in the ctrl. The foliar application of increasing concentrations of SAC significantly decreased the Cd concentration in both the shoots and roots. At 0.2 mmol·L^−1^ of SAC, the Cd concentrations in the shoots and roots decreased by 49.0% and 29.8%, respectively, compared to those of the ctrl. However, when the concentration of SAC was further increased to 0.4 mmol·L^−1^, there was no significant difference in the Cd concentrations in the rice seedling shoots and roots compared to the 0.2 mmol·L^−1^ treatment group.

As shown in Figure 1B–D, the Ca and Mg concentrations in the shoots were much higher than those in the roots. However, the concentration of Fe was much lower in the shoots than in the roots. The foliar spraying with SAC had no significant effect on the concentrations of Ca, Mg, or Fe in the roots or shoots of the rice seedlings. These results showed that spraying with SAC had no significant effect on the transport of Ca, Mg, or Fe from the roots to the shoots in rice.

As shown in Figure 1E,F, high concentrations of Mn and Zn accumulated in the nutrient storage organs of the rice seedlings. The foliar spraying with SAC significantly decreased the Mn content in the roots and shoots of the rice seedlings. Upon spraying 0.2 mmol·L^−1^ of SAC, the Mn concentrations in the roots and shoots significantly decreased by 21.5% and 16.2%, respectively. The foliar spraying with SAC had no significant effect on the Zn concentration in the roots of the rice seedlings. However, the concentration of Zn in the shoots of the rice seedlings decreased significantly after spraying with SAC. With increasing SAC concentration, the Zn concentration in the shoots also decreased gradually. When the concentration of SAC was increased to 0.2 mmol·L^−1^, the Zn concentration in the shoots decreased by 35.6% compared to that in the ctrl. When the concentration of SAC was 0.4 mmol·L^−1^, the concentration of Zn in the shoots was not significantly different compared with that in the group sprayed with 0.2 mmol·L^−1^ of SAC.

In summary, these data show that the optimum spraying concentration of SAC to decrease the translocation of Cd from the roots to shoots was 0.2 mmol·L^−1^.

### 3.2. Effect of Spraying SAC on Cd Stress in Rice Seedlings

**Figure 2:**
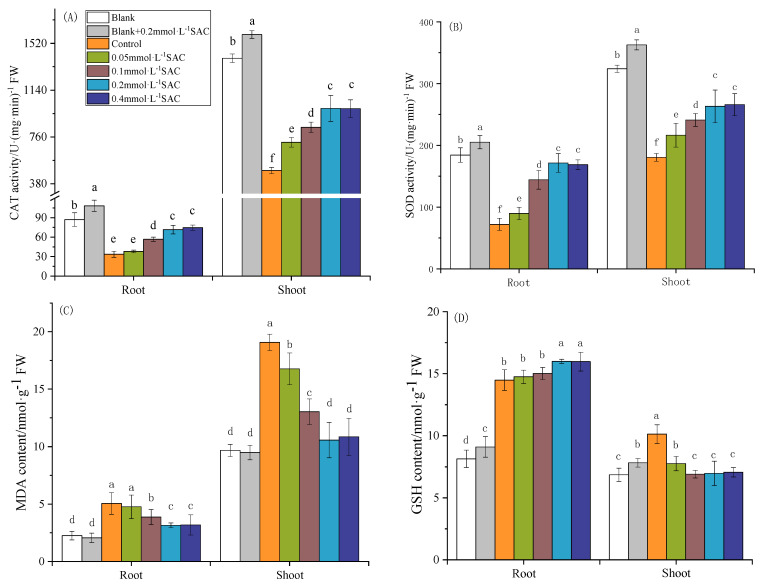
Effect of spraying SAC on Cd stress in rice seedlings: (**A**) CAT activity, (**B**) SOD activity, (**C**) MDA content, and (**D**) GSH content. a, b, c, d, e and f represent the significance of the differences in each treatment.

To study the effect of SAC on alleviating Cd stress, we measured the activities of the antioxidant enzyme, CAT and SOD as well as the MDA and GSH contents in the rice seedlings. As shown in Figure 2, the activities of CAT (Figure 2A) and SOD (Figure 2B) in the ctrl were significantly lower than those in the BK in the roots (61% and 61%, respectively) and shoots (65.2% and 44.3%, respectively) of the rice seedlings. These data indicate that the rice seedlings were severely stressed in the presence of CdCl_2_. Spraying with increasing concentrations of SAC led to an increase in the activities of the two antioxidant enzymes in the roots and shoots. Upon spraying 0.2 mmol·L^−1^ of SAC, the CAT and SOD activities reached a maximum (103.6% and 45.8% greater than in the ctrl in the shoots; 113% and 138% greater than in the ctrl in the roots, respectively). When the concentration of SAC was increased to above 0.2 mmol·L^−1^, the activities of the two enzymes did not show any further increase. After spraying the BK with 0.2 mmol·L^−1^ of SAC, the activities of CAT and SOD in the roots increased by 24.6% and 11.3%, respectively, and increased by 13.7% and 11.9% in the shoots, respectively.

As shown in Figure 2C,D, the contents of MDA and GSH in the roots of the ctrl increased by 146.4% and 77.8%, respectively, and those in the shoots increased by 97.1% and 47.5%, respectively, compared to those in the BK. After spraying with SAC, the MDA concentration in the seedlings decreased significantly. When the SAC concentration was increased to 0.2 mmol·L^−1^, the MDA concentration in the roots and shoots decreased by 37.6% and 44.6% compared to those in the ctrl. The GSH concentration in the shoots was significantly reduced by 31.1% compared with that in the ctrl upon spraying 0.2 mmol·L^−1^ of SAC. After spraying the BK group with 0.2 mmol·L^−1^ of SAC, the GSH concentration in the roots and shoots significantly increased by 11.7% and 14.1%, respectively, but it did not have a significant impact on the MDA concentration.

These data indicate that spraying with SAC alleviated the Cd stress in the rice seedlings. Furthermore, the maximum effect was achieved with a SAC concentration of 0.02 mmol·L^−1^.

### 3.3. Effect of Spraying SAC on Cd Content in CW Components of Rice Seedling Roots

**Figure 3:**
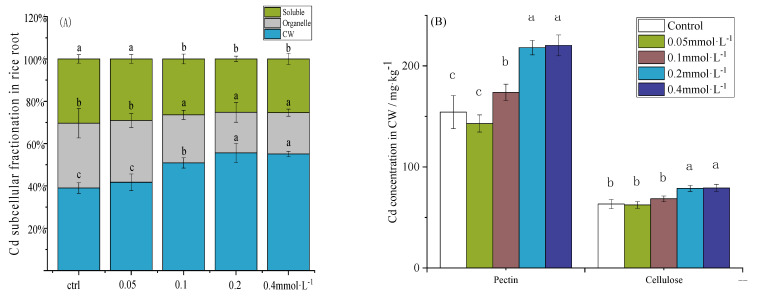

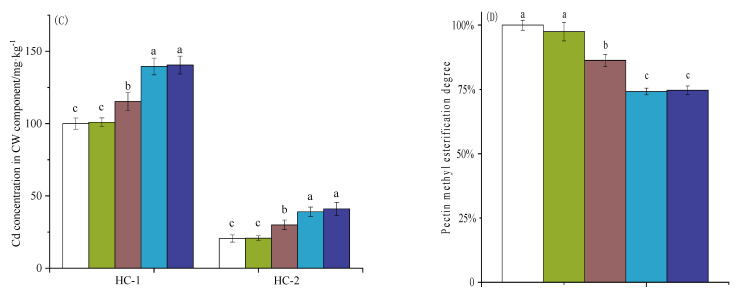
Effect of spraying SAC on Cd content in CW components in roots of rice seedlings. (**A**) Subcellular distribution of Cd in roots of rice seedlings, (**B**) Cd concentration in pectin and cellulose components of root CW, (**C**) Cd concentration in HC-1 and HC-2 components of root CW, and (**D**) methyl esterification degree of pectin component in root CW. a, b and c represent the significance of the differences in each treatment.

As shown in Figure 3A, when the spraying concentration of SAC increased, the Cd concentration in the CW components of the rice seedling roots gradually increased. At a SAC spraying concentration of 0.2 mmol·L^−1^, the Cd concentration in the CW components significantly increased by 43.5% compared to that in the ctrl. In contrast, the concentrations of Cd in the O and S components significantly decreased by 37.3% and 17.0%, respectively. When the spraying concentration of SAC was higher than 0.2 mmol·L^−1^, there was no significant change in the Cd concentration among these three components.

Figure 3B,C show that the accumulated Cd concentration in the pectin component of the root CW of the rice seedlings was the highest, followed by that in the HC-1 component. When a low concentration of 0.05 mmol·L^−1^ of SAC was sprayed, there was no significant effect on the Cd concentration in any component of the CW. As the SAC spraying concentration increased, the Cd concentrations in the pectin, cellulose, HC-1, and HC-2 in the root CWs of the rice seedlings showed a gradually increasing trend. The highest Cd accumulation was detected at a SAC spraying concentration of 0.2 mmol·L^−1^, and further increasing the SAC spraying concentration had no significant effect on the Cd concentration in any component of the root CW. Compared with the ctrl, spraying 0.2 mmol·L^−1^ of SAC significantly increased the Cd concentration in the pectin, cellulose, HC-1, and HC-2 components by 131%, 117%, 40%, and 90%, respectively.

As shown in Figure 3D, with an increase in the SAC spraying concentration, the degree of methyl esterification of the pectin components in the root CW of rice seedlings gradually decreased. Spraying 0.05 mmol·L^−1^ of SAC had no significant effect on the degree of esterification of the pectin components. However, when the concentration of SAC spraying reached 0.2 mmol·L^−1^, the degree of esterification of the pectin components decreased by 24.8% compared to that in the ctrl.

### 3.4. Effect of Spraying SAC on the Expression of Cd Uptake and Transport Genes in Rice Seedlings

**Figure 4:**
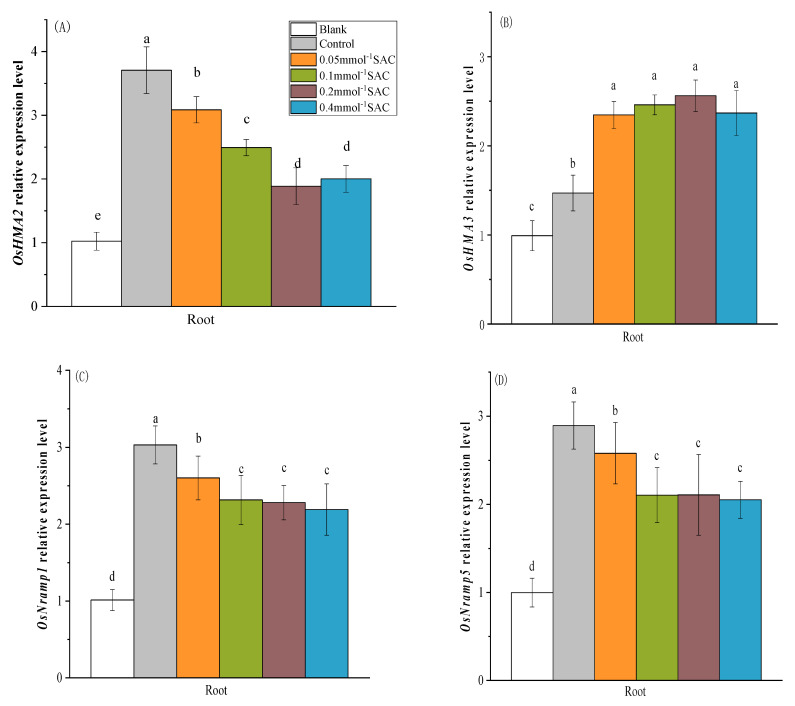
Effect of spraying SAC on the expression of Cd uptake and transport genes in rice seedlings: (**A**) OsHMA2, (**B**) OsHMA3, (**C**) OsNRAMP1, and (**D**) OsNRAMP5 relative expression levels. a, b, c, d and e represent the significance of the differences in each treatment.

A qRT-PCR assay was performed to determine the gene expression of four metal transporters, OsHMA2, OsHMA3, OsNRAMP1, and OsNRAMP5, to investigate the molecular mechanism. As shown in Figure 4, in the ctrl, the expression levels of OsHMA2, OsHMA3, OsNRAMP1, and OsNRAMP5 in the roots were increased by 269%, 48%, 199%, and 190%, respectively, compared with those in the BK. The foliar application of SAC significantly downregulated the expression levels of OsHMA2, OsNramp1, and OsNramp5. When the SAC spraying concentration reached 0.2 mmol·L^−1^, the expression levels of the three Cd transport genes were significantly reduced by 49.1%, 24.8%, and 27.2%, respectively. When the spraying concentration was increased to 0.4 mmol·L^−1^, the expression levels of the three genes did not change compared with those at 0.2 mmol·L^−1^.

However, spraying SAC significantly upregulated the expression level of the OsHMA3 gene, which significantly increased by 42.6% compared to that in the ctrl when the spraying concentration reached 0.2 mmol·L^−1^, and no significant differences were observed between spraying treatments at different concentrations.

### 3.5. Effect of Spraying SAC on NO Content in Rice Seedlings

**Figure 5:**
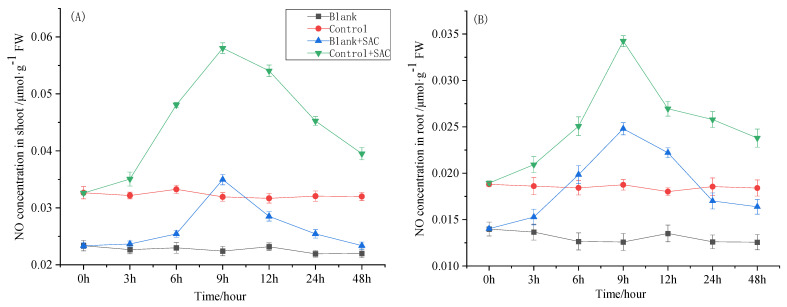
Effect of spraying SAC on NO content in rice seedlings. (**A**) Shoot (**B**) Root.

As shown in Figure 5, after spraying 0.2 mmol·L^−1^ of SAC on the BK group, the NO concentration in the seedling shoots and roots continued to increase over the 0–9 h period. The NO concentration in the shoots and roots reached its observed highest value, 1.55 and 1.96 times higher than that of the BK, 9 h after spraying SAC. Subsequently, the NO concentration in the shoots and roots gradually decreased. Under Cd stress, the changes in the NO concentration in the shoots and roots in the seedlings after spraying 0.2 mmol·L^−1^ of SAC were similar to those in the BK, being approximately 1.8 times higher than in the ctrl at the ninth hour. Cd stress also increased the NO concentration in the rice seedlings, and the NO concentration in the shoots and roots in the ctrl was approximately 1.5 times higher than that of the BK.

## 4. Discussion

Foliar spraying technology has become an important agricultural tool for reducing the Cd content in rice grains. With the rapid development of unmanned aerial vehicle (UAV) spraying technology in China, foliar spraying has become widely used in agricultural production. Our previous experiments showed that spraying SAC in the field can significantly reduce the Cd content in rice grains (Sfig1); however, the potential mechanism through which SAC spraying regulates Cd transport had not yet been reported. In this study, spraying 0.2 mmol·L^−1^ of SAC significantly reduced the Cd contents in the shoots and roots of the rice seedlings compared with that observed in the ctrl. These data indicated that spraying SAC significantly inhibited the uptake and translocation of Cd from the roots to the shoots.

Plant physiological studies [37] have shown that Cd is rapidly transported from the roots to shoots by the xylem after absorption, determining the Cd accumulation in rice shoots and grains. Takahashi et al. [12] reported that the OsHMA2 transporter is mainly responsible for transporting Zn and Cd from the roots to the shoots in rice; the inactivation of OsHMA2 decreased the root-to-shoot translocation of Cd and Zn [13]. In the present study, when 2.7 µmol·L^−1^ of Cd^2+^ was present in the culture medium, the OsHMA2 expression was significantly upregulated (by 269.2%) in the ctrl compared with that in the BK, implying that the presence of Cd^2+^ significantly induced OsHMA2 expression. However, after spraying with different concentrations of SAC, the expression level of OsHMA2 gradually decreased. Correspondingly, the Cd and Zn concentrations in the shoots of the rice seedlings decreased by 49.0% and 35.6%, respectively. These data indicated that the main molecular mechanism leading to the reduced Cd and Zn concentrations in the shoots of rice seedlings after treatment with SAC was the downregulation of OsHMA2 gene expression. Reports indicated that the Zn concentration in the shoots of rice seedlings decreased significantly in OsHMA2 gene mutant lines [13]. However, the rice seedlings grew normally. In our study, spraying with SAC significantly reduced the Zn concentration in the rice seedlings without affecting growth.

There is no specific transporter for Cd uptake in rice as it is a toxic and nonessential metal element in plants. Heavy metal ions enter plants primarily through the metal cation channels in the roots [7]. Cd is mainly transported into rice roots by the Mn transporter OsNRAMP5 [38], which is polarly localized on the distal side of both the endodermis and exodermis of rice roots. Following OsNRAMP5 knockout, the Mn and Cd contents in rice roots, shoots, and grains decreased significantly [9,38]. In addition, studies have shown that OsNRAMP1 is also involved in the absorption of Cd and Mn [10]. Therefore, an important mechanism for reducing the accumulation of Cd in rice is to decrease the absorption of Cd from the external environment into the roots. This study used qRT-PCR to detect the changes in the gene expression levels of the OsNRAMP5 and OsNRAM1 transporters after spraying with SAC. The results showed that compared with those in the ctrl, the relative expression levels of OsNRAMP5 and OsNRAMP1, encoding the Mn/Cd transporter, were downregulated by 24.8% and 27.2%, respectively, when the SAC spraying concentration was 0.2 mmol·L^−1^.

After crossing the plant cell plasma membrane, toxic Cd^2+^ is transported to the vacuole, an important plant detoxification mechanism [14]. OsHMA3 acts on the vacuole membrane of root cells and plays a crucial role in the transport of Cd into the vacuoles. Sasaki et al. [39] reported that the overexpression of OsHMA3 significantly increased Cd tolerance in rice. Similarly, Zhang et al. [11] found that the overexpression of OsHMA3 in wheat increased the sequestration of Cd in wheat roots and significantly reduced the transport of Cd from the roots to shoots and the accumulation of Cd in wheat grains. Therefore, the OsHMA3 transporter can affect Cd transport in plants by increasing the distribution of Cd to vacuoles, retaining Cd in the roots, and reducing its transport to the shoots. In the present study, we tested the expression of OsHMA3 under Cd stress. The results showed that compared with those in the BK, the relative expression levels of the Cd transporter-coding gene OsHMA3 were upregulated significantly under Cd stress conditions in the ctrl. This indicated that the rice seedling initiated its detoxification mechanism and began storing Cd in the vacuoles under Cd stress. Furthermore, spraying SAC further upregulated the expression level of OsHMA3 compared to that in the ctrl. This result indicated that spraying with SAC promoted Cd transport and segregation into the vacuoles of the rice roots, which may have further reduced Cd transport to the rice shoots.

Plant CWs consist of pectin, hemicellulose, cellulose, and other components. CWs are the first barrier to Cd entry to root cells. Various active chemical groups on CWs, such as thiol (-SH), hydroxyl (-OH), and carboxyl (-COOH), can chelate Cd, preventing Cd from entering the cytoplasm and thereby reducing its toxicity to cells. Previous reports have shown that pectin components rich in carboxyl groups play the most important role in fixing Cd to the CW [40]. Pectin biosynthesis is mainly completed in the Golgi complex and is transported to the CW in a highly methyl esterified form [41]. Pectin demethylation is primarily catalyzed by pectin methyl esterase. The generated exposed carboxyl groups of pectin could efficiently bind Cd to fix it onto the CW. Therefore, a negative correlation exists between the degree of pectin methylation and the ability of pectin to fix Cd. In addition, the hemicellulose and cellulose components can fix Cd. In this study, the Cd content in the root CW was determined first, followed by the Cd contents in the pectin, hemicellulose, and cellulose, which are CW components. The results showed that spraying SAC significantly increased the Cd content in the root CW.

The seedling stage is a key stage in the growth and development of rice. Under high concentrations of Cd^2+^ stress, plants produce several ROS that damage the cell membrane system by producing MDA and affect the activities of antioxidant enzymes, such as SOD and CAT. Plants initiate their own defense mechanisms by synthesizing large amounts of GSH to chelate heavy metal ions [30]. Subsequently, the growth and development of rice seedlings are significantly affected and are characterized by slow growth, blocked root development, and decreased biomass [42]. In the present study, the activities of CAT and SOD in the roots and shoots of the rice seedlings significantly decreased and the concentrations of MDA and GSH significantly increased in the presence of Cd in the growth media. All these parameters indicated that the rice seedlings were under severe Cd^2+^ stress. After spraying with SAC, the activities of the two antioxidant enzymes (SOD and CAT) in the shoots of the rice seedlings were significantly restored, and the MDA and GSH concentrations were significantly decreased. The changes in the SOD, CAT, and MDA in the roots were similar to those in the shoots. However, after spraying SAC, the GSH content in the roots gradually increased, while the content in the shoots showed a decreasing trend. Due to the high Cd content in the roots, even after spraying SAC, the Cd still reached about 900 mg·kg^−1^. At that time, Cd was still highly toxic to the seedlings, and the rice continued to initiate GSH synthesis. However, the Cd content in the shoots significantly decreased, resulting in a significant decrease in the synthesis of GSH in the shoots. These results indicated that spraying SAC significantly alleviated the Cd stress in the shoots of the rice seedlings. The results obtained for the shoots of the rice seedlings are consistent with reports that SAC can scavenge ROS and alleviate heavy metal stress in mammals [24].

Plant signaling systems regulate the expression of functional genes and physiological processes related to plant development. Recent studies have shown that NO is involved in regulating plant CW synthesis, Cd transporter gene expression, and responses to abiotic stress [16,17]. In plants, NO mainly originates from nitrate reduction, which is first reduced to nitrite under sequential catalysis by nitrate reductase and then further reduced to NO. Therefore, the NO synthesis process in plants is a branch of the nitrate assimilation process [15]. In this study, we measured the changes in the NO content sin the shoots and roots of rice seedlings. Under Cd stress (ctrl), the NO content in roots was approximately 1.5 times higher than that in the BK group without Cd addition throughout the testing process, which indicated that the rice seedlings started responding to abiotic stress through an increase in NO synthesis. After spraying 0.2 mmol·L^−1^ of SAC, the NO content increased rapidly during the first 9 h. At the 9 h point, the NO content in the seedlings increased to 1.8 times higher than that in the ctrl. Then, the NO content gradually decreased. These results, in combination with the downregulation data of Cd absorption and transporter gene expression and the root CW Cd content data at 72 h after spraying with SAC, demonstrated that an increased NO content regulated the expression of Cd-transport-related genes and the interception of Cd by CW compared to the ctrl.

In this paper, spraying SAC triggered the biosynthesis of NO signaling molecules. There have been reports indicating that NO signaling molecules in plants can induce the biosynthesis of H_2_S signaling molecules [16] and the plant hormone, salicylic acid [43]. They can all alleviate heavy metal stress in plants and regulate heavy metal transport. Under our experimental conditions, the generated NO may have induced the biosynthesis of other signaling molecules, but further research is still needed.

## 5. Conclusions

In this study, we found that foliar spraying with SAC increased the NO contents in the seedlings, reduced the Cd contents in the roots and shoots, and regulated the expression levels of Cd-transport-related genes in rice seedlings. The optimal spraying concentration of SAC was determined to be 0.2 mmol·L^−1^. Furthermore, spraying with SAC increased the activities of SOD and CAT in the shoots of the rice seedlings and upregulated OsHMA3 expression, promoting the segregation of Cd into vacuoles in the root cells, thus alleviating Cd stress. Additionally, spraying SAC increased the ability of the root CW to intercept Cd.

## Data Availability

The raw data supporting the conclusions of this article will be made available by the authors upon request.
